# Endoscopic versus microscopic transsphenoidal surgery in the treatment of pituitary tumors: systematic review and meta-analysis of randomized and non-randomized controlled trials

**DOI:** 10.1590/2359-3997000000204

**Published:** 2016-08-31

**Authors:** Rodrigo V. S. Bastos, Carla Maria D. M. Silva, Jose Vicente Tagliarini, Marco Antonio Zanini, Flavio R. Romero, Cesar Luiz Boguszewski, Vania dos Santos Nunes

**Affiliations:** 1 Departamento de Clínica Médica Faculdade de Medicina de Botucatu Universidade Estadual Paulista Botucatu SP Brasil Departamento de Clínica Médica, Faculdade de Medicina de Botucatu, Universidade Estadual Paulista (Unesp), Botucatu, SP, Brasil; 2 Departamento de Oftalmologia e Otorrinolaringologia Faculdade de Medicina de Botucatu Universidade Estadual Paulista Botucatu SP Brasil Departamento de Oftalmologia e Otorrinolaringologia, Faculdade de Medicina de Botucatu, Universidade Estadual Paulista (Unesp), Botucatu, SP, Brasil; 3 Departamento de Neurologia, Psiquiatria e Psicologia Faculdade de Medicina de Botucatu Universidade Estadual Paulista Botucatu SP Brasil Departamento de Neurologia, Psiquiatria e Psicologia, Faculdade de Medicina de Botucatu, Universidade Estadual Paulista (Unesp), Botucatu, SP, Brasil; 4 Departamento de Clínica Médica Universidade Federal do Paraná Curitiba PR Brasil Departamento de Clínica Médica, Divisão de Endocrinologia, Serviço de Endocrinologia e Metabologia (SEMPR), Universidade Federal do Paraná (UFPR), Curitiba, PR, Brasil

**Keywords:** Pituitary tumor, surgical endoscopy, neurosurgical procedure, review, systematic

## Abstract

We conducted a systematic review and meta-analysis of randomized and non-randomized controlled trials that compared pure endoscopic with microscopic transsphenoidal surgery (TSS) in the resection of pituitary tumors. Embase, PubMed, Lilacs, and Central Cochrane were used as our data sources. The outcomes were total tumor resection, achievement of biochemical control of functioning adenomas, hospital stay and surgery complications. The randomized trials were analyzed using the Grading of Recommendations Assessment, Development, and Evaluation (GRADE) approach. Two randomized and three prospective controlled non-randomized studies were included. Two studies, including 68 patients, evaluated total tumor resection and the meta-analysis did not show differences between the groups [RR: 1.45 (95% CI: 0.87, 2.44)]. Three studies involving 65 patients analyzed the achievement of biochemical control and no statistical difference was found [RR: 0.94 (95% CI: 0.7, 1.26)]. All five studies compared the frequency of postoperative complications between intervention and control group and meta-analysis favored for a low rate of postoperative complications in the endoscopic TSS group [(RR: 0.37 (95% CI: 0.16, 0.83)]. Due to the low evidence level and low number of observations, the results of our meta-analysis should not be viewed as a final proof of inferiority or superiority of one approach in relation to the other. More data including higher numbers of observations are needed.

## INTRODUCTION

Transsphenoidal surgery (TSS) is the treatment of choice for most functioning and nonfunctioning pituitary tumors. The microscopic technique is a traditional and well-established procedure that has been successfully applied for removal of pituitary adenomas. Most available data about the short- and long-term outcomes of TSS are derived from this modality of surgery. In the 1990s, a distinct impulse came from the otorhinolaryngologists, with the use of the endoscope in functional endoscopic sinus surgery, disclosing the pathway to the sella turcica and the endoscopic approach for resection of pituitary tumors either alone or as an adjunct to the microneurosurgery.

Microscopic TSS can be performed via sublabial or endonasal transeptal approach. The latter has a slightly smaller field of view in comparison with the former, but it has the advantages of being less painful and not requiring postoperative packing (1). In the endoscopic technique, a rigid endoscope is used to get into the sphenoid sinus and the sella turcica through both nostrils. Some experienced neurosurgeons and otorhinolaryngologists have changed to the endoscopic surgery, claiming that this procedure is safer, less invasive, and allows a wider view of the sella turcica, improving tumor resection rates (1).

Studies comparing endoscopic with microscopic TSS have produced inconsistent results, either showing no difference between them (2-4) or favoring the new technique (5,6). The main reasons for these discrepancies include comparisons of retrospective results of microscopic TSS versus prospective results of endoscopic TSS and the experience of the surgical teams with the different procedures. Thus, a careful analysis of each series reported on the literature is essential before asserting the superiority of one technique over the other.

The aim of the present study was to perform a systematic review and meta-analysis of randomized and non-randomized controlled trials that compared pure endoscopic with microscopic TSS in the resection of pituitary tumors. Our hypothesis was that endoscopic TSS would be safer and more effective than the microscopic technique in the management of pituitary tumors in experienced hands.

## MATERIALS AND METHODS

We have included randomized, non-randomized, or quasi-randomized controlled trials that were carried out to compare endoscopic TSS (intervention group) with microscopic TSS (comparison group) involving adult patients with acromegaly, Cushing’s disease, nonfunctioning pituitary adenomas (NFPA), prolactinomas resistant to dopamine agonists or other pituitary-related tumors.

The primary postoperative outcomes analyzed in our study included total tumor resection, achievement of biochemical control of functioning adenomas and frequency of postoperative complications, such as: cerebrospinal fluid (CSF) leak, temporary or permanent diabetes insipidus, hypopituitarism, hypothyroidism, hypocortisolism, sinusitis, meningitis, nasal synechiae, saddle nose, syndrome of inappropriate antidiuretic hormone secretion (SIADH), hyposmia, anosmia, blood loss, massive nasal bleeding, wound disruption and vision decline. Secondary outcomes were quality of life, costs, duration of postoperative hospital stay (days) and operative time.

We searched the following electronic databases: Embase (1980–2014), PubMed (1966–2014), Lilacs (1982–2014) and the Cochrane Central Register of Controlled Trials (CENTRAL, the Cochrane Library, issue 2014). Ongoing clinical trials on the ClinicalTrials.gov website, in conferences, hand-searches and specialists in this field were also consulted. The Medical Subject Heading (MeSH) terms used included ‘‘Acromegaly’’, “Pituitary ACTH Hypersecretion”, ‘‘Pituitary Neoplasms’’, “Hyperpituitarism”, ‘‘Endoscopic Transsphenoidal Surgery’’, “Microscopic Transsphenoidal Surgery’’. There was no language or year restriction.

Two reviewers (RVSB and JVT) independently screened the titles and abstracts identified by the literature search and the studies potentially eligible for inclusion in the review were selected for complete reading. In case of disagreements, there was a debate between the reviewers and a third party (VSN) before the final decision.

For each trial, we assigned risk of bias taking in consideration quality scores for random sequence generation, allocation concealment, blinding of outcomes and incomplete outcome data. We used the criteria described in the Cochrane Reviewers’ Handbook (7) to classify these scores in adequate (low risk of bias), unclear, and inadequate (high risk of bias).

We performed the meta-analysis using Review Manager 5.3 software. For dichotomous outcomes, relative risk was calculated with a 95% confidence interval and we expressed continuous variables as weighted mean difference along with their 95% confidence intervals. Potential causes of heterogeneity among the studies were also analyzed. The I^2^ statistic was used to measure the proportion of statistical heterogeneity for each outcome. When the data were homogeneous, we undertook a fixed-effect analysis. However, in the presence of heterogeneity (I^2^ > 0), the analysis was performed in a random-effect model. The sensitivity analysis was also performed by excluding non-randomized controlled trials.

The quality of evidence per outcome measurement was graded according to the guidelines of the GRADE (Grading of Recommendations Assessment, Development, and Evaluation) Working Group (8,9). The confidence of GRADE system decreases if studies have major limitations that may interfere on the estimates of the treatment effect (8). These limitations include risk of bias at each included study, and GRADE also considers inconsistency, indirectness, imprecision and publication bias of each evaluated outcome. Randomized studies have risk of bias when they failure to conceal allocation, failure to blind, loss to follow-up, and failure to appropriately consider the intention to treat principle (10). Inconsistency is related to heterogeneity of the results, indirectness compares whether the interventions are delivered to the adequate populations, imprecision refers to what extent our confidence in the estimated effect is adequate to support a particular decision (confidence intervals) and publication bias should be suspected when available evidence comes from a number of small studies (11,12), most of which have been pharmaceutical industry sponsored (13,14).

## RESULTS

**Figure 1:**
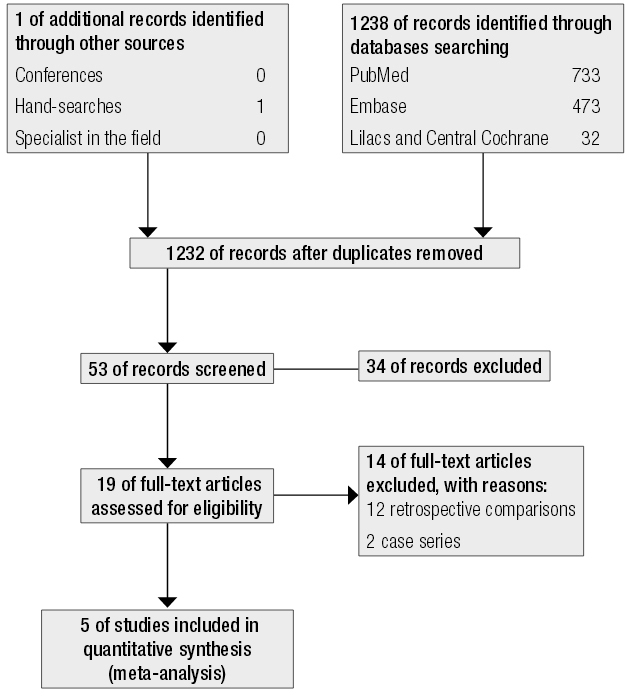
Flowchart for identifying eligible studies

Our search identified 1238 references (Figure 1). Nineteen articles were potentially eligible for inclusion in our review, and from these 12 were excluded because they involved retrospective comparisons (2,3,5,15-23) and two were excluded because they were publication of case series (24,25). Five publications were included in the final analysis (4,6,26-28).

Five studies included in the meta-analysis involved a total of 328 participants; two were randomized controlled trials (6,26) and three were non-randomized controlled trials (4,27,28). The main characteristics of the enrolled patients are presented in Table 1.

**Table 1:** Main characteristics of the enrolled patients and risk of bias in the five included studies

Study/Year	Type of study number of patients	Inclusion criteria	Outcomes	Risk of Bias			

				Randomization	Blinding of outcome assessment	Allocation	Incomplete outcome data
Jain, 2007	Randomized 10/10	Patients with pituitary adenoma	Complete tumor resection, achievement of biochemical control of functioning adenomas, postoperative complications, operative time	Appropriate (low risk)	No mentioned (unclear)	No mentioned (unclear)	No (low risk)
Enseñat, 2009	Prospective Non-randomized 25/23	Patients with pituitary adenoma	Complete tumor restriction, achievement of biochemical control of functioning adenomas, postoperative complications, hospital stay	No (high risk)	No mentioned (unclear)	No applicable	No (low risk)
Cho, 2002	Randomized 22/22	Patient with prolactinoma and with intolerance to bromocriptin	Visual field, achievement of biochemical control of functioning adenomas, postoperative complications, hospital stay, operative time	No mentioned (unclear)	No mentioned (unclear)	No mentioned (unclear)	No (low risk)
Little, 2014	Prospective Non-randomized 67/99	Patients with pituitary tumor	Hospital charges, postoperative complications, hospital stay,	No (high risk)	No mentioned (unclear)	No applicable	3 patients (low risk)
Kahilogullari, 2013	Prospective Non-randomized 25/25	Patients with pituitary tumor	Postoperative complications	No (high risk)	No mentioned (unclear)	No applicable	No (low risk)

Jain and cols. (26) randomized 20 patients with pituitary adenomas (9 NFPA, 5 acromegaly, 3 prolactinomas, 2 Cushing’s disease, 1 Nelson syndrome) to endoscopic or microscopic TSS. Most patients had macroadenoma. An endoscopic rhinologist performed the endoscopic TSS and a neurosurgeon performed the microscopic TSS; both were dedicated surgeons who have been operating for more than 15 years. Primary outcomes were complete tumor removal, mean operative time, blood loss, and CSF leak. All patients were subjected to a magnetic resonance imaging (MRI) three months after surgery that showed the same complete tumor removal rate in both groups.

Cho and Liau (6) randomized to endoscopic or microspic TSS 44 patients with prolactinoma who were operated due to intolerance to bromocriptine or visual deficits. The groups had similar baseline characteristics, the mean follow up was 3.5 years and the main outcomes were normal prolactin levels, normal menstrual cycle, no galactorrhea, visual field improvement, operative time, hospital stay and complications.

In a prospective study performed in Phoenix, Arizona, 166 patients were assigned to one of the study groups according to surgeon preference: 67 to endoscopic (43 NFPA, 7 acromegaly, 7 prolactinomas, 3 Cushing’s disease, 7 other) and 99 to microscopic TSS (57 NFPA, 13 acromegaly, 11 prolactinomas, 7 Cushing’s disease, 11 other (Rathke’s cleft cyst, meningioma, pituitary cyst) (4). One surgeon performed all microscopic TSS and another one all the endoscopic TSS. The secondary outcomes were length of stay in the hospital (days) and complications (temporary or permanent diabetes insipidus, CSF leak, hyponatremia, cardiac dysrhythmia, respiratory failure, delirium tremens, heart palpitations, vision decline, and meningitis).

Kahilogullari and cols. (28) performed a non-randomized controlled study comparing olfactory functions between 50 patients operated by endoscopic or microscopic TSS (44 pituitary adenomas (30 hormone-active and 14 NFPA), 3 fibrous dysplasia, 1 Rathke cyst, 1 chordoma, 1 hypophysitis). The Smell Diskettes Olfaction Test was applied before and 1 and 6 months after the surgery to evaluate olfactory outcomes. The authors also studied incidence of CSF leak. In the endoscopic group, they observed only two patients with hyposmia, while in the microscopic group, 13 patients developed anosmia and 5 anosmia.

Enseñat and cols. (27) performed a prospective, non-randomized study, to compare the TSS techniques in relation to the total and subtotal tumor resection, surgical complications and time of post-operative hospitalization. They included 50 patients who had sellar lesions with different grades of invasiveness of the cavernous sinus (47 pituitary adenomas, 3 Rathke cyst).

The risk of bias (random sequence generation, allocation concealment, blinding of outcome assessment and incomplete outcome data) of five included studies are presented in Table 1.

### Meta-analysis

**Figure 2:**
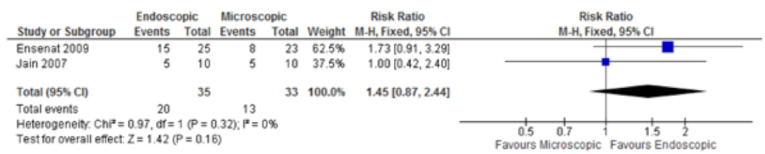
Meta-analysis of total tumor resection.

**Figure 3:**
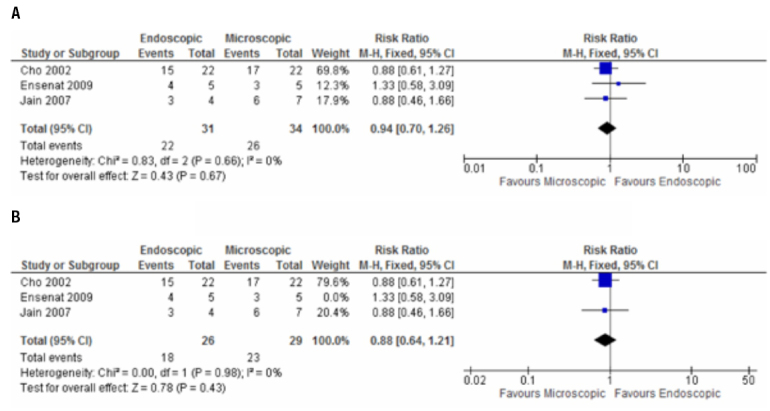
Meta-analysis of achievement of biochemical control of functioning adenomas, A-B. A. Meta-analysis of randomized and non-randomized studies. B. Meta-analysis of two randomized studies

Two studies evaluated total tumor resection (26,27); the meta-analysis did not show significant difference between the groups [RR: 1.45; (95% CI: 0.87, 2.44)] (Figure 2). Three studies (6,26,27) analyzed achievement of biochemical control of functioning pituitary adenomas and, again, no statistical difference was found between the intervention and comparison groups [RR: 0.94; (95% CI: 0.7, 1.26)] (Figure 3A).

Three studies assessed the duration of postoperative hospital stay (4,6,27), but only two mentioned standard deviation (SD) and could be included in the meta-analysis (4,27). The result was heterogeneous (92% (I^2^)), with one study favoring the intervention group and the other one showing no difference between the groups [MD: -1.26 (95% CI: -4.1, 1.59)].

**Figure 4:**
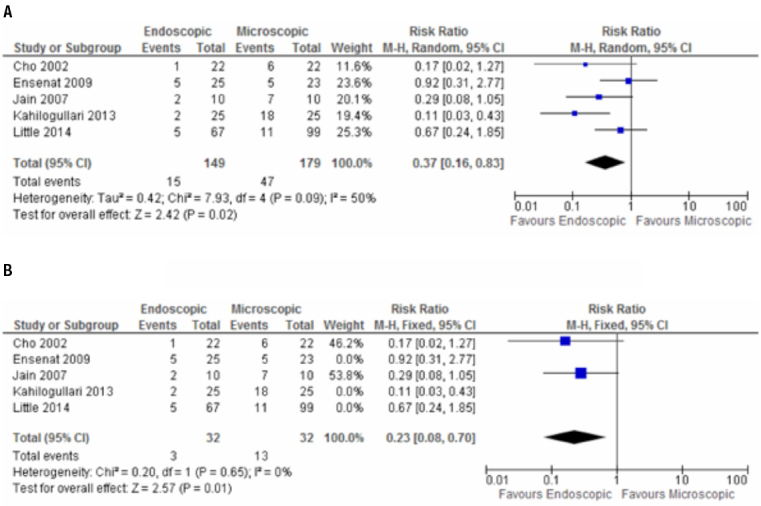
Meta-analysis of postoperative complications; A-B. A. Meta-analysis of randomized and non-randomized studies. B. Meta-analysis of two randomized studies.

All five studies compared frequency of postoperative complications between intervention and control group and our meta-analysis favored for a low rate of postoperative complications in the endoscopic TSS group [(RR: 0.37; (95% CI: 0.16, 0.83)] (Figure 4A).

Only Little and cols. (4) evaluated total hospital charges, and there was no statistical difference between the groups ($74,703 ± $15,142 for microscopic surgery, $72,311 ± $16,576 for endoscopic surgery; p = 0.33). Hospital charges did not differ significantly by category, with the exception of higher charges for pathology in the microscopic surgery group ($6, 46 ± 2,605 for endoscopic and $8,626 ± 3,650 for microscopic, p < 0.001).

Quality of life was not evaluated in the included studies. The two randomized studies evaluated time of operation and endoscopic TSS was performed faster than microscopic TSS in both of them. However, meta-analysis could not be performed in this outcome because only one study indicated SD.

### Quality of evidence

As shown in Table 2, the quality of evidence according to GRADE approach was performed only for two common outcomes in the two randomized studies (postoperative complications and achievement of biochemical control of functioning adenomas) (6,26).

**Table 2:** GRADE approach for primary outcomes

Patient outcomes	Quality of assessment					Summary of findings		

	Risk of bias	Inconsistency	Indirectness	Imprecision	Publication Bias	Relative effect (95% CI)	RRR	Quality
Postoperative complications 2 RCTs (55)	Serius (-1)	No inconsistency	Direct	No imprecision	Serius (-1)	RR 0.23 (0.08-0.7)	77%*	++, low quality
Achievement of biochemical control of functioning adenomas, 2 RCTs (55)	Serius (-1)	No inconsistency	Direct	Serious (-1)	Serius (-1)	RR 0.88 (0.64-1.21)	12%**	+, very low quality

* In favor of interventional group. ** In favor of control group. Low quality evidence: the authors are not confident in the effect estimate and the true value may be substantially different from it. Very low quality evidence: the authors do not have any confidence in the estimate and it is likely that the true value is substantially different from it.

Despite that the randomized studies did not have lost of follow-up, the GRADE approach for our outcomes “postoperative complications” and ”achievement of biochemical control of functioning adenomas” was necessary to rate down for the risk of bias, because only the study by Jain and cols. (26) mentioned randomization process, while concealment allocation was not clear in neither of them. The sensitivity analysis of these outcomes excluding non-randomized controlled trials showed a RR was 0.23 (CI: 0.08-0.70) and 0.88 (CI: 0.64-1, 21) respectively (Figures 3B and 4B). There was no need to rate down for inconsistency, as no heterogeneity at the meta-analysis results was observed. Similarly, it was not necessary to rate down for indirectness for both outcomes. Imprecision was rated down for “achievement of biochemical control of functioning adenomas”, because the 95% CI did not exclude a RR of 1. Concerning publications bias, rate down of quality was necessary due to small number of randomized controlled trials. Quality of evidence for postoperative complications was low and for achievement of biochemical control of functioning adenomas was very low.

## DISCUSSION

With recent advances on endoscopic sinus surgery, the use of endoscope to reach the sella turcica has emerged as an alternative method for resection of pituitary tumors (29). Additionally, it has been suggested that the endoscopic surgery, in comparison with microscope approach, may be associated with more gross-total tumor resection, shorter hospital stay, shorter operation time, lower estimated blood lose, low complication rates and better endocrine outcomes (30). However, there is no clear evidence that this concept is true and available results from published studies are still controversial.

Atkinson and cols. (2) published a retrospective comparison between these two surgical modalities. They found shorter anesthesia time, hospital stay and less blood loss with the endoscopic technique. However, they found no difference in postoperative complications or hormonal disease control. The authors concluded that success rate was more related to the surgeon experience than to the surgery technique itself.

Considering nonfunctioning pituitary adenomas (NFPAs), Messerer and cols. (3) published a retrospective study and demonstrated a greater total tumor resection rate with the endoscopic technique (74% vs 50%, p = 0.002). On the other hand, Cappabianca and cols. (5) conducted a comparison between a prospective group of patients operated with endoscope and a retrospective group operated with microscope and found no difference between them. However, hospital stay was shorter in the endoscopic group (3.1 ± 0.4 vs 6.2 ± 0.3 days, p < 0,001).

Choe and cols. (30) performed a study that included 17 acromegalic pacients; eight operated by the microscopic approach from 1997 to 2004 and nine by the endoscopic approach from 2004 to 2007. In the endoscopic group, eight patients had full tumoral resection and obtained criteria for cure, while the same outcomes were seen in only three patients in the microscopic group.

Our systematic review included only prospective randomized and non-randomized controlled studies comparing the two TSS techniques, because retrospective studies are more likely to provide biased information. In addition, in almost all published retrospective comparisons, the microscopic surgeries were done chronologically before the endoscopic surgeries. As TSS efficiency is correlated to the surgeons’ ability and experience, the superiority of one specific technique over the other may be attributed much more for the accumulated time of neurosurgeon experience than the technique itself.

Our primary outcome of total tumor resection was only possible to be evaluated in two studies (26,27). Jain and cols. (26) considered tumor complete excision by direct visualization of sella turcica (sella turcica was inspected for residual tumor with a 308 endoscope) and Enseñat and cols. (27) defined total resection by no visualization of tumor remnants in a MRI carried out three months after surgery. Although the meta-analysis of this endpoint showed a RR of 1.45 favoring intervention group, with an I^2^ = 0% it was not possible to exclude RR of 1.0 (CI: 0.87-2.44) in the 95% CI.

Achievement of biochemical control of functioning adenomas was evaluated in three studies (6,26-27). Cho and Liau (6) considered normalization of prolactin levels as hormonal control in patients with prolactinomas. Enseñat and cols. (27) used GH levels less than 1 ng/mL after oral glucose tolerance test with normal IGF-1 as criteria for cure in acromegaly, prolactin levels < 30 mg/dL in women and < 15 mg/dL in men for cure in prolactinomas and normal urinary free cortisol for cure in Cushing’s disease. Jain and cols. (26) did not mention the criteria used to determine the biochemical control. The meta-anlysis of this endpoint showed a risk ratio of 0.94, favoring control group. However, the 95% CI did not exclude a RR of 1 (0.7-1.26), even with sensitivity analysis excluding non-randomized studies. These findings demonstrated a serious imprecision, and based on GRADE approach, the quality of evidence was very low, with a substantial chance that the true value is considerably different from what we observed.

The safety of the two TSS approaches was evaluated by comparison of postoperative complications rates in the five studies included in our review. The analysis was related to all complications reported at postoperative time, since it was not possible to determine the risks for each complication separately in the studies. The meta-analysis showed a relative risk of 0.37 (95% CI: 0.16-0.83), resulting that endoscopic surgery reduced in 63% the chance of surgical complications in relation to the microscopic technique, but with an I^2^ = 50%. We have tried to solve this heterogeneity performing a sensitivity analysis with the exclusion of non-randomized controlled trials. The significance was maintained in favor of endoscopic group, with a RR of 0.23 (CI: 0.08-0.70) and I^2^ = 0. However, applying GRADE approach, the quality of evidence was not high, due to small number of randomized studies and the lack of information related to process of randomization and concealment allocation.

Other systematic reviews comparing endoscopic and microscopic TSS have been previously published (31-35). Two (6,26) studies included in our meta-analysis also appear in some of the previous published systematic reviews (32-33), while the other three (4,27,28) are included only in our study. Some reports used either Medline or Medline and Embase electronic databases to retrieve published studies (33-35). In contrast with our review, all of them had language restriction (English) and predominantly included retrospective studies in their analysis. Moreover, most of them did not specify the number of reviewers who participated on quality assessment process, the potential causes of heterogeneity among the studies and the random-effects model. In three systematic reviews, the authors compared the results of endoscopic TSS in a particular study with those of microscopic TSS performed in another unrelated study (31-34).

In summary, based on prospective controlled studies, our review did not find a significant difference between endoscopic and microscopic TSS in relation to pituitary tumor resection rates and achievement of biochemical control of functioning pituitary adenomas. Postoperative complications seem to be less frequent with the endoscopic procedure. However, the quality of evidence for these outcomes was low or very low according to GRADE system. Due to this low evidence level and low number of observations, the results of our meta-analysis should not be viewed as a final proof of inferiority or superiority of one approach in relation to the other at this moment. New randomized trials with larger number of patients, more precise comparisons, and long-term outcomes, are needed.
